# Putting Public Health Ethics into Practice: A Systematic Framework

**DOI:** 10.3389/fpubh.2015.00023

**Published:** 2015-02-06

**Authors:** Georg Marckmann, Harald Schmidt, Neema Sofaer, Daniel Strech

**Affiliations:** ^1^Institute of Ethics, History and Theory of Medicine, Ludwig-Maximilians-University Munich, Munich, Germany; ^2^Department of Medical Ethics and Health Policy, Center for Health Incentives and Behavioral Economics, Perelman School of Medicine, University of Pennsylvania, Philadelphia, PA, USA; ^3^Centre of Medical Law and Ethics, The Dickson Poon School of Law, King’s College London, London, UK; ^4^Institute for History, Ethics and Philosophy of Medicine, Centre for Ethics and Law in the Life Sciences (CELLS), Hannover Medical School, Hannover, Germany

**Keywords:** public health practice, health policy, ethics, program evaluation, ethical theory

## Abstract

It is widely acknowledged that public health practice raises ethical issues that require a different approach than traditional biomedical ethics. Several frameworks for public health ethics (PHE) have been proposed; however, none of them provides a practice-oriented combination of the two necessary components: (1) a set of normative criteria based on an explicit ethical justification and (2) a structured methodological approach for applying the resulting normative criteria to concrete public health (PH) issues. Building on prior work in the field and integrating valuable elements of other approaches to PHE, we present a systematic ethical framework that shall guide professionals in planning, conducting, and evaluating PH interventions. Based on a coherentist model of ethical justification, the proposed framework contains (1) an explicit normative foundation with five substantive criteria and seven procedural conditions to guarantee a fair decision process, and (2) a six-step methodological approach for applying the criteria and conditions to the practice of PH and health policy. The framework explicitly ties together ethical analysis and empirical evidence, thus striving for evidence-based PHE. It can provide normative guidance to those who analyze the ethical implications of PH practice including academic ethicists, health policy makers, health technology assessment bodies, and PH professionals. It will enable those who implement a PH intervention and those affected by it (i.e., the target population) to critically assess whether and how the required ethical considerations have been taken into account. Thereby, the framework can contribute to assuring the quality of ethical analysis in PH. Whether the presented framework will be able to achieve its goals has to be determined by evaluating its practical application.

## Background

Public health ethics (PHE) is a relatively new field of applied ethics, and is concerned with the moral implications of a diverse range of activities aiming to protect or improve population health. It is an interdisciplinary field that has to take into account both moral *and* factual considerations, in health policy and health sciences. PH practice differs considerably from medical practice: while the latter is primarily concerned with the health of individual patients, the former focuses on the health of populations. Protecting and promoting health and preventing diseases constitute the primary objectives of PH interventions, rather than treating sick individuals. Often, collective efforts of the community are required to achieve PH goals. Last but not least, it has been questioned whether the prevailing liberal approach to medical ethics is appropriate for the field of PH (1). It is therefore widely acknowledged that ethical inquiries in PH need a different approach than traditional biomedical ethics (2, 3).

To arrive at transparent, consistent, and justified results, ethical analysis in PH should follow a clearly defined methodological approach. Any framework for PHE thereby has to meet at least two fundamental requirements: (1) as a tool for normative inquiry, the framework must be based on an *explicit ethical justification*. This is a requirement of any ethical analysis: normative claims about what is morally acceptable must be justified by an underlying ethical theory or at least an explicit ethical approach. A transparent normative basis allows those who act upon the analysis (e.g., PH professionals) and those who are affected by the analysis (e.g., the target population) to assess the validity of the resulting claims. (2) A framework for PHE should provide practical guidance for the various people working within or related to the field of PH. It therefore should include a *methodological approach* that relates the general normative considerations such as ethical norms, values, and principles and the available empirical evidence to concrete PH interventions, programs, or policies.

Several PHE frameworks have been developed over the last years [for some recent reviews, see Ref. (4–6)]. They differ with respect to their theoretical foundations, the selection of normative principles, and the practical guidance (6). So far, none of these frameworks has won universal approval as the go-to ethical framework in the field of PH – which may also be due to the fact that PHE is still a rather new field of inquiry. In addition, none provides a fully elaborated account of the two necessary ingredients of a PHE framework noted above, comprising both a clearly defined *ethical foundation* and a *methodological approach*. Some frameworks present a set of ethically relevant questions or points to consider without explicitly defining the normative basis, i.e., the underlying ethical principles and their justification (2, 7). Other frameworks comprise ethical principles for evaluating PH interventions, but fail to explain in more detail how these principles should be applied to evaluate particular interventions (3, 8). Childress et al. present a set of general moral considerations, i.e., an explicit normative foundation, and address some practical questions related to specifying and weighting the general moral considerations and resolving conflicts among them (9). While this certainly enhances the practical utility, it does not comprise a more comprehensive methodological approach that can guide – step by step – the ethical planning, conduct, and evaluation of PH policy.

In this article, we first outline what the preconditions and advantages of a *systematic* framework for PHE are. Building on and combining prior work in the field, we then present a systematic framework for addressing ethical issues in the field of PH that tries to satisfy both the foundational and methodological requirements. The framework comprises (1) an explicit *normative foundation* with five substantial ethical criteria and seven procedural conditions guiding a fair decision process, and (2) a six-step *methodological approach* for applying the ethical criteria and conditions. We have developed the framework primarily to provide practical guidance to those who want to analyze the ethical implications of PH practice including academic ethicists, health policy makers, health technology assessment bodies, and – last but not least – PH professionals.

## What Makes a Framework for PHE Systematic?

In a PHE framework, both the selection of relevant ethical norms and principles and their application to a public heath program should be performed in a systematic way. The *selection* can be considered systematic if it follows a defined methodological approach to identify a comprehensive list of relevant ethical norms and principles that should be considered in every ethical analysis of PH practice [see e.g., Ref. (3, 8, 9)]. Any modification of the normative foundation by changing, adding, or omitting ethical considerations must be explicitly justified. Furthermore, the *application* of the ethical norms and principles to a certain PH program or issue can be considered systematic if it follows an explicitly defined process [see e.g., Ref. (2, 7, 9)]; again, any deviation from the procedure should be justified.

Using a systematic approach to PHE has several advantages:

(1) It reduces the risk that the evaluation neglects relevant ethical considerations or an important methodological step (10).
(2) It allows an explicit assessment of the process quality of a PHE analysis by checking whether the relevant norms and principles have been considered and whether the required methodological steps have been completed.
(3) It enables health policy makers, PH professionals, and members of the target population to assess whether the relevant norms and principles have been considered (11), whether and why they have been modified, and whether all methodological steps have been completed.
(4) It promotes a more explicit understanding among PH students and professionals what it means to assess the ethical implications of PH interventions.

Overall, a systematic framework to PHE has the potential to increase the quality of ethical analysis and reflection in the field of PH.

## The Normative Foundations

As it is one of the fundamental goals of PHE to provide normative guidance in the field of PH, the framework must be grounded in an ethical theory or at least ethical approach that provides a justification of the selected principles and norms. However, there is intractable disagreement about which ethical theory is correct. Moral philosophy is characterized by a multitude of competing approaches that differ significantly in their justificatory strategies and PHE frameworks vary considerably with respect to their philosophical foundations (6). According to consequentialist theories, for example, the action or policy that has the best consequences is morally right (in utilitarianism the action that maximizes overall utility), irrespective of the resulting distribution. According to deontological theories, by contrast, moral obligations and individual rights determine which action is ethically mandated (e.g., to respect individual autonomy). As a consequence, the results of ethical analyses will vary considerably depending on the underlying ethical theory.

An alternative approach that explicitly acknowledges the complexity of normative orientations in modern pluralistic societies is the coherentist model of justification, which has been introduced as “reflective equilibrium” by John Rawls (12). In our view, it is also the most promising model for PHE (9). Unlike classical ethical theories, coherentism does not build on a single foundational moral principle, but rather starts with considered judgments, i.e., moral convictions and beliefs that we hold in our everyday life, and develops a *coherent* framework by specifying, testing, and revising them. The goal is a “reflective equilibrium” of theoretical assumptions, moral principles, and judgments about single cases. The four principles beneficence, non-maleficence, respect for autonomy, and justice, for example, are internationally recognized as a coherentistically justified set of moral principles for the field of biomedicine (13).

The principles that have been developed from considered judgments represent *prima facie* binding moral norms that must be followed unless they conflict with equally strong or stronger obligations. Moreover, they provide only general ethical orientations that require further content to give guidance in concrete cases. Thus, in application, the principles have to be specified and – in case of conflict – balanced (see below: methodological approach).

A coherentist model of justification has several advantages: despite unresolved foundational issues, it allows us to find consensus on the level of *prima facie* binding mid-level principles, since they build on our everyday moral convictions and are compatible with various ethical justifications. At the same time, it makes moral controversies more transparent, since they can be analyzed as conflicts between principles with different weights. Identifying precisely the type of ethical conflict is often the first step toward a solution.

Based on the coherentist model of justification, we have developed a normative foundation for PHE that contains five substantive ethical criteria (see Substantive Normative Criteria) and seven procedural conditions (see Procedural Conditions for a Fair Decision Process).

### Substantive normative criteria

Table 1 presents the substantive normative criteria that should guide ethical analysis in PH based on a coherence approach of justification (see above). They are linked to the specific characteristics of the field of PH, thereby taking into account that PH focuses on populations rather than individuals, works preventively rather than curatively, and usually requires action at the population rather than the individual level (1). Many ethical principles and considerations relevant for PH have already been elaborated over the last several years [cf. the reviews Ref. (4–6)]. As “considered judgments” about what is morally important in PH, they constitute the basic ingredients of our coherentist approach and are reflected – explicitly or implicitly – in our list of normative criteria. They are justified by more basic ethical principles including maximizing health benefits, preventing harm, respecting autonomy, or promoting justice. The order of the criteria is determined by the sequence of their application. First of all, the benefit of the intervention has to be established. Without an expected benefit, the intervention should not be implemented and there is no need to apply the other criteria. After assessing expected benefits and potential harms of the intervention, implications on individual autonomy, distributive justice, and efficiency can be evaluated. It is important to realize that this set of criteria constitutes only the starting point of any ethical analysis. Before applying the criteria, evaluators have to assess whether all criteria are relevant to the PH intervention or policy decision, whether further criteria have to be taken into consideration and whether the criteria require further specification for the application domain.

**Table 1:** **Substantive normative criteria for ethical analysis in public health**.

Normative criteria	
1	*Expected health benefits for the target population*
	• Range of expected effects (endpoints)
	• Magnitude and likelihood of each effect
	• Strength of evidence of each effect
	• Public health (practical) relevance of effects
	• Incremental benefit compared to alternative interventions
2	*Potential harm and burdens*
	• Range of potential negative effects (endpoints)
	• Magnitude and likelihood of each negative effect
	• Strength of evidence for each negative effect
	• Public health (practical) relevance of the negative effects
	• Burdens and harms compared to alternative interventions
3	*Impact on autonomy*
	• Health-related empowerment (e.g., improved health literacy)
	• Respect for individual autonomous choice (e.g., possibility of informed consent, least restrictive means)
	• Protection of privacy and confidentiality (e.g., data protection)
4	*Impact on equity*
	• Access to the public health intervention
	• Distribution of the intervention’s benefits, burdens and risks
	• Impact on health disparities
	• Need for compensation?
5	*Expected efficiency*
	• Incremental cost-benefit/cost-effectiveness ratio
	• Strength of evidence for expected efficiency

#### 1. What are the expected health benefits of the intervention for the target population?

An ethical evaluation of a PH intervention must start with assessing its expected benefit. This requires defining the goals of the intervention with the range of expected effects. These can be surrogate endpoints, e.g., the identification of cancer in its early stages or more patient-oriented endpoints, e.g., lowering the cancer-specific mortality rate. The magnitude and likelihood of the effect should be quantified (e.g., reduction of the morality rate for breast cancer from 4/1000 to 3/1000 in the next 10 years through mammography screening for healthy 30-year-old women) (14). In addition, the validity of the available evidence is relevant. Are the underlying studies randomized-controlled trials or retrospective cohort studies? How adequately have the studies been implemented and published [e.g., selective reporting of study findings on the health-related effects of smoking (15))? Besides *internal* validity (the credibility of the results for the study sample), the *external* validity of the demonstrated effect is also relevant. The external validity concerns the credibility of the results outside of the study sample and thus indicates how generalizable the results are. Only if a *relevant* health-related effect can be demonstrated or justified on the basis of sufficiently *valid* study results, does it make sense to speak of “benefits” of a certain PH intervention. The intervention-specific, health-related benefit should be higher than the potential benefits of alternative interventions, thereby providing an *additional* benefit for the target population.

An expected benefit can seem plausible even if the underlying evidence is not of the highest desirable internal and external validity. In this case, it is necessary to explicitly state the reasons for the lack of suitable data and the arguments why it nevertheless seems appropriate to implement the intervention. This transparency is a necessary prerequisite for dealing appropriately with the frequently uncertain demonstration of benefits in the field of PH (16, 17).

The necessity to review alternative PH interventions to achieve the same goal is not just an imperative of instrumental rationality and the principle of benefit maximization, but also allows us to identify any alternatives that might be ethically less problematic – e.g., by being less restrictive on individual autonomy (see third principle).

#### 2. What are the potential burdens and harms of the intervention?

Oftentimes, beneficial PH interventions are associated with social and health risks and burdens (e.g., false positive findings with consecutive unnecessary interventions in the case of cancer screening). For this reason, it is important to assess not only potential benefits but also potential harms. Potential harm should be assessed for those directly and indirectly affected and be compared with the expected benefit for the target population to determine the net-benefit. Analogous to the expected benefit, the magnitude, likelihood, and scientific validity of the potential harm need to be assessed [cf. Ref. (18)]. It is one of the central goals of the ethical assessment to recommend suitable measures for reducing the – often unavoidable – risk of harm for the individual as much as possible.

In summary, (i) the practical relevance of the different endpoints (e.g., decreased cancer-related mortality vs. improved early detection of cancer), (ii) the magnitude and likelihood of the effects, and (iii) the scientific validity of the demonstrated effects plays a crucial role in the ethical evaluation of a PH intervention. The controversy among experts on the benefits and harms of mammography screening exemplifies how differently these three aspects can be assessed in a single intervention (19) and how these differences affect PH decision making.

After balancing benefits and harms of the intervention, we can determine whether overall there is a “net-benefit” or “net-harm.” Only if there is a (sufficiently valid) actual or expected net-benefit does it make sense to continue the ethical evaluation.

#### 3. How does the intervention affect the autonomy of the individuals in the target population?

The ethical principle *respect for autonomy* is relevant to PHE in two ways. First, PH interventions can and should (if possible) improve the health literacy and competence of the target population (20–22). For this purpose, it is necessary to provide, among other things, high-quality information about the type of intervention and its potential benefits and harm, adapted to the needs of people with different knowledge, capabilities and ways of accessing information (23, 24). Second, in light of the usually unavoidable burdens and risks, individuals should generally be able to decide themselves about their participation in a certain PH program after being sufficiently informed (informed consent). If individual informed consent to participation is not possible (e.g., tab water fluoridation), there should be a democratically legitimate public decision process about the implementation of the PH intervention.

If certain PH goals can only be achieved effectively by influencing or even restricting individual freedom of choice (e.g., incentive systems, legal obligations, or quarantine interventions), this requires a special justification. In particular, it has to be demonstrated that the PH goal cannot be achieved with a less restrictive or less manipulative intervention (25). As a general rule, restrictions should be minimized (9). For example, before legally mandating a PH intervention, there should be an attempt to achieve sufficient participation by non-coercive incentives. The fact that a less restrictive intervention might forfeit a potential health benefit for the population is not *per se* an argument for more restrictive interventions. The expected health benefit rather has to be balanced against the potential social harm (restriction of freedom, protection of privacy, or stigmatization).

#### 4. Impact on equity: how are benefits and burden distributed?

Public health interventions often have an impact on the distribution of health outcomes and therefore the opportunities that citizens are offered in a society (26). For reasons of *equity*, therefore, all people who might benefit should have equal access to a given PH intervention. Both financial and non-financial barriers to access have to be taken into account. In addition, the distribution of potential benefits and harm has to be examined. PH interventions should contribute to reducing existing health inequalities. For example, interventions can be tailored to the needs of health-disadvantaged groups (while avoiding possible negative social consequences like stigmatization).

When PH interventions accept a potential harm for certain subgroups to achieve a significant expected benefit for another subgroup, strategies to compensate for these risks have to be considered for the sake of compensatory justice. For example, people placed under quarantine need to be given appropriate psychological support and their captivity should be alleviated as well as possible beyond the regular standard of care in hospitals. Another example concerns health professionals exposed to an increased risk of infection by their patient contact during a pandemic (e.g., SARS): they should be compensated by an independent fund to cover their illness or absence from work (27).

#### 5. Expected efficiency: what are the costs and opportunity costs of the intervention?

In the light of limited public resources, the *efficiency* of a PH intervention has to be assessed. This requires determining the incremental cost–benefit ratio, i.e., the ratio between additional costs and additional benefit compared to alternative interventions (if available). The type of benefit and harm entering into the net-benefit of the PH interventions has to be explicitly defined. As with the potential benefit and harm, the internal and external validity of the efficiency assessment have to be evaluated. Determining the *incremental* cost–benefit ratio presupposes reviewing the alternative (if any) strategies to achieve the same PH goals.

### Procedural conditions for a fair decision process

Since PH interventions have an impact on the well-being and autonomy of individuals and often require collective efforts, they should be implemented by a *legitimate* decision-making authority within a fair process. Even reasonable and fair-minded people often come to different conclusions in the face of complex moral deliberations. Among other things, this is due to the fact that many evaluations – e.g., of health-related benefits – can only be made on the basis of substantial visions of a good or fulfilled life. How can we make *legitimate* decisions under these conditions of moral controversy? Norman Daniels argues that we have to supplement the general substantive principles of justice with a fair decision process that “holds decision makers accountable for the reasonableness” of their decisions (26). “Accountability for reasonableness” requires four procedural conditions of fairness: transparency (*publicity condition*), reasonable explanation (*relevance condition*), openness for revision (*revision and appeals condition*), and the regulation of adherence to the other three conditions (*regulative condition*) (26). We suggest adding consistency, participation, and managing conflicts of interest (28–30), so that any ethical analysis of PH interventions has to assess how far the seven conditions for a fair decision process described in Table 2 are met. Further conceptual research is necessary to develop quality criteria for the practical implementation of the seven conditions (e.g., what determines a high quality, reasonable explanation?). Further empirical research is necessary to evaluate the feasibility as well as the intended and unintended effects of the seven conditions (31). The results may help to determine more specific guidelines on the adequate implementation of the seven procedural conditions in the practice of PH (32–34).

**Table 2:** **Conditions of a fair decision process**.

Conditions for a fair decision process		
1	Transparency	Decision process including database and underlying normative assumptions should be transparent and public
2	Consistency	Application of the same principles, criteria and rules across different public health interventions → equal treatment of different populations
3	Justification	Decisions should be based on relevant reasons, i.e., based on the normative criteria for PHE (Table 1)
4	Participation	Populations affected by the PH intervention should be able to participate in the decision about the implementation
5	Managing conflicts of interest	Decisions about PH interventions should be organized so as to minimize any existing and manage any remaining conflicts of interests of decision makers
6	Openness for revision	Implementations of PH interventions should be open for revision (e.g., if data basis changes or certain aspects have been neglected)
7	Regulation	Voluntary or legal regulation should guarantee that these conditions for a fair decision process are met

## Methodological approach to PHE

After having laid out substantive ethical criteria and conditions for a fair decision process, we now present a step-by-step methodological approach that shall guide the ethical evaluation of a given PH intervention in the different phases of its development, implementation, and evaluation.

### 

#### 1. Description of the public health intervention

Any ethical analysis must start with a thorough characterization of the PH intervention, the context in which it will be applied, and possible alternative interventions to achieve the PH goal that might minimize potential negative impact on PH, individual autonomy, equity, or efficiency.

#### 2. Specification and modification of the normative criteria

After describing the PH intervention, the normative basis of the evaluation needs a critical review: do the normative criteria (cf. Table 1) require further specification or even supplementation for the PH intervention? The practical relevance of each principle should be clarified, starting with a concrete statement of the content and scope of the principle for the PH intervention at hand. Different policy makers or evaluators may arrive at different specifications with potentially different results in the analysis. While this cannot be eliminated completely, using this explicit framework at least requires the evaluators to explicitly define and justify the specifications so that the underlying sources of disagreement become transparent – and thereby open to revision.

In practice, the five normative criteria (Table 1) are often given unequal consideration. For example, an investigation might focus more on balancing expected benefits (criterion 1) with the restrictions of autonomy (criterion 3), while neglecting equity implications (criterion 4). In the ethical debates on mammography screening, equity is often overlooked, despite the screening’s high costs (35) and the well-known disparities in breast cancer outcomes between racial and ethnic groups (36). Similarly, many criticisms of national pandemic plans make some effort to apply criteria 1–3 but do not explicitly consider criteria 4–6 (37, 38).

#### 3. Evaluation of the public health intervention using the specified criteria

In the third step, each of the specified normative criteria is used to evaluate the PH intervention. The evaluators must ask, for example: what are the expected benefits of the intervention? What are the program’s implications for the autonomy of members of the target population? A step-by-step assessment can reveal currently unresolved controversies and identify the need for further conceptual or empirical studies.

#### 4. Synthesis: overall evaluation of the public health intervention

The fourth step requires compiling each assessment from the previous step into an overall evaluation of the PH intervention. This involves identifying conflicts between the criteria and balancing the conflicting ethical obligations. Balancing requires finding convincing reasons why one criterion or the other should prevail. Being explicit about the reasons that determine the relative weights of the conflicting criteria creates transparency and allows a revision of the balancing by challenging the underlying reasons. For example, there might be good reasons to doubt the validity of the information considered in a particular case or competing information might be available.

The balancing of conflicting ethical obligations shall be illustrated by two examples:

Example 1: in considering a quarantine of a tuberculosis patient, we have to balance respect for autonomy (criterion 3) and protecting others from the risk of a transmitted tuberculosis infection (here: criterion 1). The severity and high likelihood of the anticipated harm to others could be a good reason to assign more weight to protecting others than to the freedom of the infected patient.
Example 2: despite empirical evidence indicating that influenza vaccination of health care personnel (HCP) in long-term care facilities may reduce the residents’ all-cause mortality and morbidity (39), vaccination rates remain rather low thus raising the question whether mandatory vaccination policies should be implemented. Here, the ethical conflict lies between the expected benefits for the long-term care residents (criterion 1) on the one side and the potential burdens and risks by the influenza vaccination for the HCP (criterion 2) and the HCP’s restriction of freedom of choice (criterion 3) on the other side. To balance the conflicting criteria, we have to assess the relative weight of the arguments (40): the benefit for the target population – 5 prevented deaths per 100 residents in one study (41) – seems to be rather large compared to the burdens and risks for the HCP due to the influenza vaccination and the restriction of freedom of choice. However, the available studies could not prove a significant effect on the primary outcome, i.e., a reduction of the mortality and morbidity caused by a laboratory proven influenza infection (39), which somewhat weakens the beneficence-based arguments in favor of a mandatory vaccination policy (for more details see Ref. (42)].

The latter example points to another important ethical consideration in the synthesis: before implementing a PH intervention that involves a conflict between the normative criteria, it is important to carefully look for alternative strategies to achieve the PH goal that are ethically less challenging. For example, if a PH intervention is particularly effective but requires a significant restriction of individual autonomy, it should be investigated whether a less restrictive intervention could lead to satisfactory results, perhaps at the price of a somewhat reduced effectiveness. Before implementing a mandatory influenza vaccination policy for HCP, for example, it has to be shown that information or incentive based programs have failed to reach sufficiently high vaccination rates to effectively protect the elderly residents (42, 43), especially in light of the somewhat limited evidence on the vaccination’s *specific* effect on mortality and morbidity in the target group.

#### 5. Generating recommendations

In most cases, the overall ethical evaluation will not result in a clear-cut rejection or endorsement of the PH intervention, but rather in a stronger or weaker recommendation, for example, to implement or – in the cases of a negative evaluation – forgo the intervention (see Table 3). Rather, it will identify various aspects and conflicts that have to be considered from an ethical perspective. In these cases, the evaluation should be translated into recommendations on how to maximize the intervention’s expected benefits and minimize the expected costs (e.g., expected social and health-related harms, restrictions of autonomy). For example, the recommendation concerning influenza vaccination of HCP could be the following: before implementing mandatory programs, further evidence on the vaccination’s specific effects and the proof that information and incentive based programs have failed are required (42). And if mandatory vaccination policies are considered, the HCP should be involved in the decision-making process (cf. criteria 4, Table 2).

**Table 3:** **Methodological approach for putting PHE into practice**.

	Step	Task
1	Description	Describe the goals, methods, target population, etc., of the PH program
2	Specification	Specify or supplement (if necessary) the five normative criteria for the PH intervention
3	Evaluation	Evaluate the PH intervention based on each of the 5 normative criteria (cf. Table 2)
4	Synthesis	Balance and integrate the 5 single evaluations of step 3 to arrive at an overall evaluation of the PH intervention
5	Recommendation	Develop recommendations for the design, implementation, or modification of the PH intervention
6	Monitoring	Monitor and re-evaluate the ethical implications in regular time intervals

#### 6. Monitoring

After successful implementation, any PH program should be followed-up and monitored in regular intervals to assess (1) whether the ethical evaluation was adequate, (2) whether there are new ethical issues arising, and (3) whether the recommendations are followed and whether they are effective in assuring an ethically appropriate execution of the PH program. For example, vaccination prioritization plans for a pandemic with “magnitude of risk” as one prioritization criterion should be re-evaluated when empirical data about the “real” risks of the pandemic are available. Or: mandatory vaccination policies should be evaluated whether they really have an additional benefit on the mortality and morbidity of elderly long-term care residents compared to voluntary programs. Furthermore, HCP’s attitudes toward the mandatory policy should be investigated by socio-empirical studies to assess how the HCP feels about the infringement of their autonomy.

Another example for the demand of monitoring the follow up and the effects of ethical recommendations is the following: there is a broad consent that mammography screening becomes more ethical if participants are adequately informed about potential benefits and harms of the screening procedure itself. The ethical analysis, however, should not stop with the recommendation to inform adequately but should be bound to the necessity of quality assessments with respect to the information process and its results (24, 44).

## Limitations

We have developed the framework primarily to provide *practical guidance*. The transparent, systematic approach will enable those who implement a PH intervention and those affected by it (i.e., the target population) to critically assess whether and how the required ethical considerations have been taken into account. Thereby, the framework can contribute to assuring the quality of ethical analysis in PH. The results of the evaluation can then be the basis for political decisions on several levels in the health care system and society about the implementation of PH interventions. While it is not the primary goal of the framework to provide guidance for these political processes, some of the ethical requirements will also apply: especially, the conditions of a fair decision process (cf. Table 2) should also be met in the political sphere of decision making – which currently is often not the case.

Whether the presented framework will be able to achieve its goals has to be determined by practical application: are all necessary normative considerations concerning substantive justification and procedural fairness included in the framework? Does the methodological approach provide a useful tool for evaluators of PH practice? Applying the framework to further examples of PH interventions will shed more light on its strengths and weaknesses. The framework itself requires critical monitoring by scholars and practitioners in the field of PH.

## Summary

There is an increasing need for assessing the ethical implications of PH practice. While several approaches have been published over the last decade, none of them give a complete account of both the normative foundation and the methodological approach. Based on a coherentist model of justification, we set out here a systematic framework for ethical analysis in PH that includes (1) an explicit normative foundation with five substantial criteria and seven procedural conditions and (2) a six-step methodological approach for applying the normative considerations to concrete PH interventions. Thereby, the framework explicitly ties together ethical analysis and empirical evidence: ethical consideration is not merely an “add-on” to empirically data. Rather, normative questions about the effectiveness, benefits, or harms of a PH intervention can only be answered by reference to the evidence from empirical studies. In this respect, the framework strives for evidence-based PHE.

## Author Contributions

GM and DS conceived of the manuscript, GM, HS, NS, and DS all made substantial contributions to the intellectual content and participated in drafting and revising the manuscript. They all read and approved the final manuscript and agree to be accountable to all aspects of the work.

## Conflict of Interest Statement

The authors declare that the research was conducted in the absence of any commercial or financial relationships that could be construed as a potential conflict of interest.
